# Generation of high affinity ICAM-1-specific nanobodies and evaluation of their suitability for allergy treatment

**DOI:** 10.3389/fimmu.2022.1022418

**Published:** 2022-11-09

**Authors:** Ines Zettl, Tatiana Ivanova, Mohammed Zghaebi, Marina V. Rutovskaya, Isabella Ellinger, Oksana Goryainova, Jessica Kollárová, Sergio Villazala-Merino, Christian Lupinek, Christina Weichwald, Anja Drescher, Julia Eckl-Dorna, Sergei V. Tillib, Sabine Flicker

**Affiliations:** ^1^ Division of Immunopathology, Institute for Pathophysiology and Allergy Research, Center for Pathophysiology, Infectiology and Immunology, Medical University of Vienna, Vienna, Austria; ^2^ Institute of Gene Biology, Russian Academy of Sciences, Moscow, Russia; ^3^ Department of Otorhinolaryngology, Medical University of Vienna, Vienna, Austria; ^4^ A.N.Severtsov Institute of Ecology and Evolution, Russian Academy of Sciences, Moscow, Russia; ^5^ Division of Cellular and Molecular Pathophysiology, Institute for Pathophysiology and Allergy Research, Center for Pathophysiology, Infectiology and Immunology, Medical University of Vienna, Vienna, Austria; ^6^ Cytiva Europe GmbH, Freiburg, Germany

**Keywords:** ICAM-1, nanobody, VHH, allergy, high affinity

## Abstract

The nasal cavity is an important site of allergen entry. Hence, it represents an organ where trans-epithelial allergen penetration and subsequent IgE-mediated allergic inflammation can potentially be inhibited. Intercellular adhesion molecule 1 (ICAM-1) is highly expressed on the surface of respiratory epithelial cells in allergic patients. It was identified as a promising target to immobilize antibody conjugates bispecific for ICAM-1 and allergens and thereby block allergen entry. We have previously characterized a nanobody specific for the major birch pollen allergen Bet v 1 and here we report the generation and characterization of ICAM-1-specific nanobodies. Nanobodies were obtained from a camel immunized with ICAM-1 and a high affinity binder was selected after phage display (Nb44). Nb44 was expressed as recombinant protein containing HA- and His-tags in *Escherichia coli (E.coli)* and purified *via* affinity chromatography. SDS-PAGE and Western blot revealed a single band at approximately 20 kDa. Nb44 bound to recombinant ICAM-1 in ELISA, and to ICAM-1 expressed on the human bronchial epithelial cell line 16HBE14o- as determined by flow cytometry. Experiments conducted at 4°C and at 37°C, to mimic physiological conditions, yielded similar percentages (97.2 ± 1.2% and 96.7 ± 1.5% out of total live cells). To confirm and visualize binding, we performed immunofluorescence microscopy. While Texas Red Dextran was rapidly internalized Nb44 remained localized on the cell surface. Additionally, we determined the strength of Nb44 and ICAM-1 interaction using surface plasmon resonance (SPR). Nb44 bound ICAM-1 with high affinity (10^-10^ M) and had slow off-rates (10^-4^ s^-1^). In conclusion, our results showed that the selected ICAM-1-specific nanobody bound ICAM-1 with high affinity and was not internalized. Thus, it could be further used to engineer heterodimers with allergen-specific nanobodies in order to develop topical treatments of pollen allergy.

## Introduction

Pollen allergy is considered one of the most common IgE-mediated hypersensitivity disorders and represents a global health and economic burden (1–3). By crossing respiratory and/or ocular surface epithelial cell layers, airborne allergens trigger IgE-mediated degranulation of underlying effector cells and cause allergic inflammation in sensitized patients (4–7). Local damage to epithelial barriers by factors such as tobacco smoke, diesel exhaust and also viral infections facilitates trans-epithelial allergen penetration, thereby increasing submucosal allergen concentrations (8, 9). Consequently local and systemic allergen-specific IgE levels are boosted, contributing to increased allergic inflammation and exacerbation of allergic disease (10–13). Since complete avoidance of pollen allergens is in most cases not feasible, a wide range of oral and topical drugs for symptomatic relief are currently available. In particular, topically active corticosteroids, antihistamines and mast cell stabilizers applied on nasal or conjunctival epithelium are widely used (14–16). In parallel, strategies to prevent loss of epithelial barrier integrity and to eventually avoid allergen entry have been pursued with great effort (17, 18). The application of nasal filters to stop or at least reduce inhalation of pollen grains has also been reported, and such devices are recommended as supplements to, but not substitutes for pharmacological measures (19, 20). Another innovative experimental approach demonstrated that topically administered allergen-specific antibodies could trap pollen allergens at epithelial surfaces and efficiently block allergen penetration of the epithelial barrier (21). To provide sustained presence of allergen-specific antibodies on the apical side of airway epithelial cells, they were immobilized to intercellular adhesion molecule 1 (ICAM-1) by ICAM-1-specific antibodies. ICAM-1 was selected as an anchor for these bispecific antibody conjugates, because it is highly expressed on epithelial cells of allergic patients and thus an attractive target molecule (22, 23). The rationale for this elegant bispecific antibody-based concept was to generate a preventive and efficacious treatment directly applied at the entry sites of airborne allergens without the common side effects reported for symptomatic treatments (20). The proof of principle study used chemical conjugates of monoclonal antibodies (21). However, to produce defined antibody constructs at reasonable expenses, smaller molecules generated at lower costs in *Escherichia coli (E.coli)* gathered interest. Accordingly, nanobodies (V_HH_ domains) derived from heavy chain-only antibodies of camelids were shown to be versatile tools for diagnosis and treatment of various diseases and, more recently, for immunoassay development in the field of type I allergy (24–30). In addition, nanobodies have similar antigen affinities as monoclonal antibodies, are chemically and physically very stable, and have a simple structure (*i.e.*, single domain antibody) (31, 32). These facts suggest that nanobodies might be promising tools for antibody-based allergy treatment. Here we report the generation, isolation and evaluation of ICAM-1-specific nanobodies. Endocytosis of ICAM-1 engaged with ICAM-1-specific nanobodies was studied in particular, because this biological pathway is commonly exploited to achieve delivery of therapeutic agents. Of note, efficient uptake of therapeutics *via* ICAM-1 has been mainly reported for multivalent ICAM-1-specific ligands and antibodies (33–37). While ICAM-1-mediated endo- and transcytosis of drugs is suitable for cancer treatment, we aimed to generate ICAM-1-specific nanobodies that remain bound to the epithelial cell surface in order to immobilize captured allergens and prevent their uptake through the mucosal barrier. Combined with a previously characterized nanobody specific for the major birch pollen allergen Bet v 1 (38), these nanobodies represent the basis for engineering bispecific nanobody formats for topical treatment of birch pollen allergy.

## Material and methods

### ICAM-1, antibodies and cell lines

Recombinant human ICAM-1 (Cat. No. ADP4), corresponding to the extracellular domains 1-5 was obtained from R&D Systems (Minneapolis, MO, USA). All antibodies used for ELISA, Western blot, flow cytometry and immunofluorescence staining were summarized in **Table S1**. The bronchial epithelial cell line 16HBE14o-, derived from human bronchial surface epithelial cells (Prof. D. C. Gruenert, University of California, San Francisco, USA) was used as surrogate for respiratory epithelium. Cells were cultured at 37°C under humid conditions and 5% CO_2_ in Minimal Essential Medium with Earle’s Salt (MEM, Gibco, Life Technologies, Carlsbad, CA, USA) supplemented with 10% HyClone Fetal Bovine Serum (FBS, Cytiva, Uppsala, Sweden), 100 U/mL Penicillin and 100 µg/mL Streptomycin (Gibco). Prior to use cell culture flasks were treated with coating solution (LHC basal medium (Gibco) supplemented with 100 µg/ml Bovine Serum Albumin (BSA, Roth, Karlsruhe, Germany), 30 µg/mL Collagen (BD Biosciences, San Jose, CA, USA) and 1 µg/mL human Fibronectin (BD Biosciences)) to ensure optimal growth conditions. For splitting, cells were washed with HBS (20 mM HEPES, 122 mM NaCl, 0.02% Glucose, 6.3 mM Na_2_HPO_4_, 0.002% Phenol Red, pH 7.4, sterilized by filtration (0.22 µm)) and detached from the surface with PET (1% Polyvinylpyrrolidone (Sigma-Aldrich, St. Louis, MO, USA), 0.02% EGTA (Sigma-Aldrich), 0.02% Trypsin-EDTA (Gibco), in HBS). The cell suspension was then centrifuged for 5 min at 1,200 rpm and the pellet was resuspended in fresh culture medium.

### Camel immunization with ICAM-1, construction of a cDNA-VHH library and selection of ICAM-1 binders

**Figure 1:**
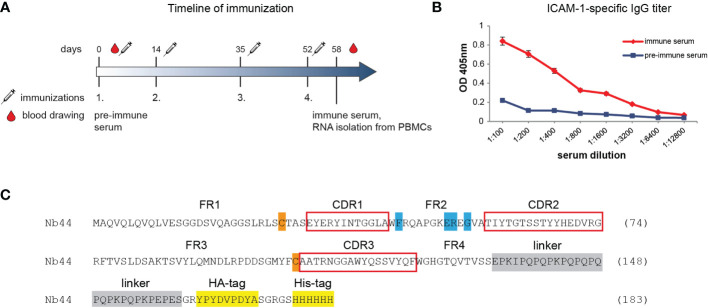
Generation of ICAM-1-specific nanobody Nb44. **(A)** shows the timeline of immunization. Intervals of injections with ICAM-1 as well as of obtaining pre-immune and immune sera are indicated (in days). The time point of isolating peripheral blood mononuclear cells (PBMCs) for generating a VHH-cDNA library is marked. **(B)** Reactivity of IgG antibodies specific for ICAM-1 in pre-immune serum (taken on day 0) and in immune serum (taken on day 58 after four injections with ICAM-1) was determined by ELISA. OD values (y-axis) correspond to the amount of bound IgG antibodies and are shown as mean of duplicates with a variation of less than 5%. Displayed data are representative of 2 independent experiments. **(C)** Amino acid sequence of Nb44. Framework regions (FR) 1-4 and complementarity determining regions (CDR, red boxes) 1-3 are indicated. Hallmark VHH amino acid substitutions (V37F, G44E, L45R, W47G) are marked in blue, cysteine residues forming the intradomain disulfide bridge are labeled in orange, the linker sequence is marked in gray and tag sequences (HA-tag, His-tag) are highlighted in yellow.

All animal work was carried out in accordance with the National Standard of the Russian Federation GOST R 53434-2009 and with approval from the Commission on Bioethics (formed in May 3, 2017) on February 2, 2018 (registration number 17) in the Severtsov Institute of Problems of Ecology and Evolution. The camel (*Camelus bactrianus*) used for immunization was kept at the Center for Collective use “Live Collection of Wild Mammals”, and immunizations were performed at the Scientific-Experimental Base “Chernogolovka” of the Severtsov Institute of Problems of Ecology and Evolution at the Russian Academy of Sciences (Chernogolovka, Russia). Before the immunization (day 0), blood was taken for later comparisons of pre-immune with immune sera (**Figure 1**). The immunization process consisted of 4 subcutaneous injections (each time the camel was injected at 4-5 sites in the upper body and neck region) over a period of 52 days. For each injection, 200 µg of recombinant ICAM-1 were used, and equal volumes of GERBU adjuvant LQ (Gerbu Biotechnik, Heidelberg, Germany) were added to the antigen preparations immediately before administration. Six days after the final immunization (day 58), 150 mL of blood were taken to measure ICAM-1-specific IgG titers and to isolate peripheral blood mononuclear cells (PBMCs) to construct a cDNA-VHH-library. Equal volumes of Phosphate-buffered Saline (PBS) containing Heparin (100 U/mL) and EDTA (3 mM) were added to the blood sample to prevent clotting. ICAM-1-specific IgG levels were analyzed with ELISA. Briefly, ELISA plate (MaxiSorp, Nunc, Roskilde, Denmark) wells were coated with ICAM-1 (2 µg/mL), blocked with 1% BSA in PBS and incubated with either immune or pre-immune sera diluted from 1:100 to 1:12,800 in PBS. Bound IgG was detected with HRP-conjugated rabbit anti-camel IgG serum (previously obtained by Tillib et al., unpublished) and 2,2’-azino-bis(3-ethylbenzothiazoline-6-sulfonic acid) (ABTS, Sigma-Aldrich). Optical density (OD) was measured at 405 nm (reference wavelength: 495 nm) using a Multiscan EX microplate reader (Labsystems, Helsinki, Finland).

The preparation of a cDNA-VHH library and selection of ICAM-1-specific binders by phage display was performed as previously described (38). Those clones that showed the strongest ICAM-1 reactivity in ELISA were sequenced. The selected DNA sequence (Nb44) was subcloned into the expression vector pHEN6 (39) (kindly provided by Prof. S. Muyldermans, Vrije Universiteit Brussel, Belgium) containing the pelB leader sequence for periplasmic expression, the long camel IgG hinge region (as a linker) and HA-tag and His-tag at the C-terminus for detection and purification purposes.

### Determination of DNA sequences corresponding to nanobodies

DNA was prepared (NucleoBond Xtra Maxi plus, Macherey-Nagel, Düren, Germany) and the nanobody coding sequence was confirmed by custom DNA sequencing (Eurofins Genomics, Ebersberg, Germany). The DNA sequence has been submitted to the GenBank database (www.ncbi.nlm.nih.gov/genbank) and translated (ExPASy, Swiss Institute of Bioinformatics, Geneva, Switzerland).

### Expression and purification of Nb44

*E. coli* XL1-Blue competent cells (Agilent Technologies, Santa Clara, CA, USA) were transformed with pHEN6 plasmid encoding the nanobody sequence and grown on LB-Agar plates with 100 µg/mL Ampicillin and 1% Glucose. Single colonies were picked and inoculated in 4 mL 2x YT medium containing 100 µg/mL Ampicillin and 1% Glucose at 37°C. Overnight cultures were transferred to 200 mL fresh medium (with 80 µg/mL Ampicillin and 0.1% Glucose) and grown until an OD600nm of 0.5 – 0.7 was reached. Expression was induced with 0.2 – 0.3 mM Isopropyl-β-D-thiogalactopyranosid (IPTG, Roth) overnight at 28°C. Cultures were centrifuged (3,500 rpm, 15 minutes, 4°C) and soluble nanobodies were extracted from the periplasm by resuspending and incubating the pellet in 4 mL TES buffer (50 mM Tris (pH 8.0), 0.5 mM EDTA, 20% Sucrose, 10 mM Imidazole, 100 µg/mL PMFS, 5 mM β-Mercaptoethanol). Following incubation for 30 minutes on ice, 6 mL 10 mM Tris (pH 8.0) with 1 mM MgCl_2_ were added for 30 minutes on ice. The suspension was centrifuged (14,000 rpm, 30 minutes, 4°C) and nanobodies in the supernatant were purified *via* HIS-Select^®^ Nickel Affinity Gel (Sigma-Aldrich). Purified nanobodies were dialyzed against PBS containing 10 mM Imidazole and protein concentration was measured by determining the absorption at 280 nm (DeNovix DS-11 FX+ spectrophotometer, Wilmington, DE, USA). Molecular mass, extinction coefficient and theoretical isoelectric point were calculated with the ProtParam tool by ExPasy.

Size and purity of Nb44 were analyzed by SDS-PAGE and Western blot. Nb44 was loaded onto a 14% polyacrylamide gel under reducing (by adding 5% β-Mercaptoethanol and heating to 95°C for 5 minutes prior to loading) and non-reducing conditions. Gels were either stained with Coomassie Brilliant Blue (InstantBlue^®^, Abcam, Cambridge, UK) or the separated nanobody samples were blotted on a nitrocellulose membrane (0.2 µm, Amersham Protran, GE Healthcare, Chicago, IL, USA). The membrane was blocked with buffer A (40 mM Na_2_HPO_4_, 0.6 mM NaH_2_PO_4_, 0.5% BSA, 0.5% Tween 20, 0.05% NaN_3_) and Nb44 was detected with a mouse anti-HA-tag antibody (1:5,000 in buffer A) coupled with HRP for 2 hours at room temperature. Bound antibodies were detected with HRP-substrate 3,3′-Diaminobenzidine (DAB, 0.6 mg/mL, Roth) in 50 mM Tris/HCl (pH 7.3).

### Reactivity of Nb44 to ICAM-1

5 µg/mL of ICAM-1 and the control protein BSA were coated onto ELISA plates for 1 hour at 37°C. Wells were washed with PBS containing 0.05% Tween20 (PBST), saturated with 1% BSA in PBST and incubated with purified Nb44 diluted to 1 µg/mL in 0.1% BSA in PBST for 1 hour at 37°C or overnight at 4°C. Wells were washed and bound nanobodies were detected with mouse HRP-labeled anti-HA-tag antibody (1:4,000 in 0.1% BSA/PBST). The color reaction was obtained with HRP substrate ABTS (1 mg/mL). ODs were measured at 405 nm (reference wavelength: 495 nm) on a TECAN Infinite F50 microplate reader (Männedorf, Switzerland) and are shown as means of triplicates ± standard deviation (SD).

### Deglycosylation of ICAM-1

ICAM-1 was deglycosylated with the enzyme Peptide-N-Glycosidase F (PNGase F) (New England Biolabs (NEB), Ipswich, MA, USA), according to manufacturer’s instructions. First, ICAM-1 (2 µg) was incubated with denaturation buffer (0.5% SDS, 40mM DTT; NEB) (total volume of 10 µL) for 10 minutes at 100°C. Then, 2 µL 10% NP-40 (NEB), 2 µL 10x Glycobuffer 2 (500 mM sodium acetate pH 7.5, NEB), 1 µL PNGase F and ddH2O to a total volume of 20 µL were added and incubated for 1.5 hours at 37°C. For the negative control, 1 µL ddH2O instead of PNGase F was added. Reaction mixtures were separated in SDS-PAGE under reducing conditions with a 12.5% polyacrylamide gel and stained with Coomassie Brilliant Blue.

### Affinity measurements using surface plasmon resonance

All used reagents were from Cytiva. Affinity measurements with ICAM-1 as ligand and Nb44 as analyte were conducted on a Biacore 3000 (Cytiva) at 25°C. ICAM-1 was immobilized on the surface of a CM5 chip (Cytiva) using amine coupling chemistry (activation with 1:1 mixture of 1-Ethyl-3-(3-dimethylaminopropyl)carbodiimide and N-Hydroxysuccinimide for 7 minutes at a flow rate of 5 µL/min). ICAM-1, diluted in 10 mM acetate buffer pH 4.5 according to a pH scout experiment, was injected to reach a level of 250 response units (RU) resulting in a maximal response of 100 RU for Nb44. The surface was deactivated with 1 M Ethanolamine for 7 minutes at a flow rate of 5 µL/min. The reference flow cell was left empty, *i.e.*, was only activated and deactivated as described above. To determine the binding affinity for Nb44 to ICAM-1, multi-cycle kinetic (MCK) assays were carried out repeatedly. Two-fold increasing concentrations of Nb44 in HBS-EP buffer (0.01 M HEPES, 0.15 M NaCl, 3 mM EDTA, 0.005% vol/vol surfactant P20 (pH 7.4)) ranging from 0.05 nM to 24 nM were injected at 30 µL/min. The association phase was 8 minutes and the dissociation phase (recorded under permanent buffer flow) was 30 minutes. Blank runs for double referencing were recorded using HBS-EP buffer and the chip surface was regenerated by injecting 10 mM glycine (pH 2) twice for 30 seconds at 30 µL/min. Dissociation constants (K_D1_, K_D2_) and rate constants (on-rates: k_a1,_ k_a2_; off-rates: k_d1,_ k_d2_) were calculated with BIAevaluation software 3.2 (Cytiva) using a heterogeneous ligand model. Experiments with Nb44 as ligand and ICAM-1 as analyte were performed on a Biacore T200 (Cytiva) at 25°C. Using the His Capture Kit (Cytiva), anti-His-tag antibodies were immobilized on the surface of a CM5 chip S series (Cytiva) with standard amine coupling strategy and Nb44 diluted in HBS-EP buffer was captured at a level of approximately 40 RU to achieve a maximal response of 100 RU for ICAM-1. Three independent single-cycle kinetic (SCK) runs and MCK runs were performed. For SCK, 2-fold increasing concentrations of ICAM-1 (7.8 nM - 125 nM) were successively injected for 2 minutes at a flow rate of 30 µL/min, and finally one dissociation phase was recorded for 15 minutes. For MCK, 2-fold increasing concentrations of ICAM-1 (3.9 nM - 250 nM) were injected for 2 minutes at a flow rate of 30 µL/min, each immediately followed by a dissociation phase for 15 minutes. In both settings, blank runs were recorded using HBS-EP buffer only and regeneration was achieved by injecting 10 mM Glycine (pH 1.5) for 60 seconds at 30 µL/min. Experimental data were fitted with a heterogeneous ligand model and K_D_s, k_a_s and k_d_s were calculated using Biacore T200 Evaluation software 3.2 (Cytiva). T-values (parameter divided by standard error of the parameter) are given for each fitted parameter value indicating reliability of the respective parameter value. With a T-value >100 a parameter is considered reliable.

### Flow cytometry

16HBE14o- cells (2 x 10^5^ cells per well) were seeded in V-bottomed 96-well plates (Greiner, Kremsmünster, Austria), washed with 0.5% BSA/PBS and blocked with 10% mouse serum (Thermofisher, Waltham, MA, USA) in 0.5% BSA/PBS for 20 minutes. Cells were washed and incubated with Fixable Viability Dye eFluor^®^ 780 (for live/dead staining) and with 20 µg/mL Nb44 or a Bet v 1-specific nanobody as isotype control (equipped with the same linker and tags as Nb44) diluted in 0.5% BSA/PBS. An ICAM-1-specific monoclonal mouse antibody was used as positive control. Isotype controls were added to the panel design to eliminate non-specific staining. After incubation of 20 minutes and another washing step, cells were stained with PE-conjugated anti-His-tag antibody or Alexa Fluor 647-conjugated anti-mouse antibody for 20 minutes. After a final washing step cells were analyzed on a BD LSRFortessa (BD Biosciences), acquiring 20,000 cells per sample in triplicates, and were evaluated with FlowJo Software (version 10, FlowJo LCC, Ashland, USA). To optimize acquisition settings, compensation beads (UltraComp eBeads, Invitrogen, Carlsbad, CA, USA) were used. All incubation steps were performed in the dark at either 4°C or 37°C.

### Immunofluorescence staining and fluorescence microscopy

16HBE14o- cells (2 x 10^4^ cells per well) were seeded on ibiTreat tissue culture-treated 8 well µ-slides (Ibidi GmbH, Munich, Germany) and grown for 1 day to reach 70-80% confluency. Cells were washed 3 times with DPBS (Gibco) and then, Nb44 or an isotype control (the before mentioned Bet v 1-specific Nb), diluted to 50 µg/mL in MEM (Gibco) supplemented with 1% BSA, was added to the cells for 30 minutes, 4 hours or 24 hours at 37°C. In control incubations, the nanobodies were omitted. Simultaneously, lysine-fixable, Texas Red-conjugated Dextran (70kDa, 10 mg/mL, Thermofisher) was added to all wells to load the endolysosomal pathway. Cells were fixed with 4% formaldehyde solution (Invitrogen) for 40 min at room temperature and remaining aldehyde groups were quenched with 50 mM ammonium chloride (in PBS) for 10 minutes. Unspecific binding sites were blocked with 3% BSA in DPBS (blocking buffer) for 30 minutes. To detect intracellular nanobodies, cells were permeabilized with 0.05% saponin added to the blocking buffer. To visualize the nanobodies, Alexa Fluor^®^ 488-labeled anti-His-tag antibody diluted 1:60 in blocking buffer (with or without saponin) was added for 1 hour at room temperature. In some control incubations, this secondary antibody was omitted and cells were incubated only with the respective blocking buffer. Cell nuclei were stained with 25 µM DRAQ5 in DPBS. In between each step and after the last one, cells were washed with DPBS. Cells were stored in DPBS at 4°C until imaging. Confocal images (30 minutes incubations) were acquired using UltraVIEW ERS Confocal Imager (Perkin Elmer, Waltham, USA) connected to a Zeiss Axiovert 200 microscope fitted with a 63x/1.4 oil objective lens (Plan-Apochromat, Zeiss, Oberkochen, Germany). Pictures were digitized and processed by Volocity software (Version 5.5., Perkin Elmer). Fluorophores were excited at 488 nm (Alexa Fluor 488), 561 nm (Texas Red) or 640 nm (DRAQ5), using a 488/561/640 multiline argon/krypton laser. Wide field fluorescence images (4h and 24h incubations) were fitted with a 63x/1.4 oil objective lens (Plan-Apochromat, Zeiss) using the Zeiss Axiovision SE64 Software Package (Version 4.9.1.0) and an AxioCam MRc5. Filter sets used for the acquisition of the fluorochromes Alexa Fluor 488, Texas and DRAQ5 were Zeiss 10, 45 and 60, respectively. Representative images were further edited with Adobe Photoshop (Version 12.0.4) using identical conditions for positive and negative controls.

## Results

### Generation of a cDNA-VHH-library and isolation of ICAM-1-specific nanobodies

Four-step immunization of a camel with recombinant human ICAM-1 corresponding to the extracellular domains 1-5 was carried out as described in the method section and summarized in **Figure 1A**. PBMCs to isolate RNA for the construction of the cDNA-VHH library were obtained 6 days after the fourth vaccination. At this time point, the animal had enhanced ICAM-1-specific IgG antibody levels as determined by ELISA (**Figure 1B**). After cloning the cDNA-sequences encoding the whole repertoire of VHHs from heavy chain-only antibodies into a phage-displayed library, three rounds of panning were conducted, ultimately leading to 73 most enriched clones. Of these, 6 clones with the strongest reactivity (as determined by ELISA) were chosen and sequenced. According to sequence analyses, all clones were identical and therefore designated as one clone, *i.e.*, Nb44. Detailed analysis of the isolated binder revealed four characteristic amino acid substitutions (from hydrophobic to more hydrophilic amino acids: V37F, G44E/K, L45R, W47G) that discriminate VHH sequences from conventional VH sequences (**Figure 1C**). These substitutions were found in the framework 2 corresponding to the site of the interface between VH and VL in conventional antibodies (**Figure 1C**). The generated VHH sequence possessed only two conserved cysteine residues (framework 1 and 3, **Figure 1C**), which form the intradomain disulfide bond that is characteristic for the Ig fold (both in VHs and VHHs). This disulfide bond is essential for the stability of VHHs (40). The sequence did not contain any additional cysteines in complementarity determining regions (CDRs), which is often the case in VHH sequences (41). The sequence data of Nb44 have been submitted to the GenBank database under accession number ON950079.

### Expression and purification of Nb44 specific for ICAM-1

**Figure 2:**
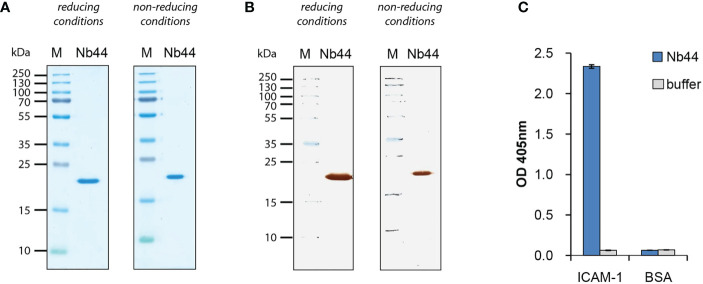
Expression and purification of nanobodies. **(A)** Coomassie Brilliant Blue-stained SDS-PAGE of ICAM-1-specific nanobody (Nb44) under reducing and non-reducing conditions and **(B)** nitrocellulose-blotted Nb44 under reducing and non-reducing conditions, detected with HRP-coupled mouse anti-HA antibody. Lane 1: protein molecular mass marker protein molecular mass marker (M), lane 2: purified Nb44, 1µg. Molecular masses are indicated on the left margin in kDa. **(C)** Reactivity of Nb44 to plate-bound ICAM-1 and control protein BSA. OD values (y-axis) correspond to the amount of bound nanobodies and are shown as means of triplicates ± SD. Data shown are representatives of 4 independent experiments.

Nb44 was expressed with a C-terminal linker and two tags (HA- and His-tag) in *E. coli* and purified *via* His-tag affinity chromatography. The theoretical isoelectric point is 6.70 and the calculated molecular mass is 20.33 kDa which includes the nanobody (around 15 kDa) and the linker with two tags (around 5 kDa combined). Analysis by SDS-PAGE (**Figure 2A**) and Western blot (**Figure 2B**) showed purified Nb44 as a clear single band at approximately 20 kDa determined under both reducing and non-reducing conditions. Reactivity tests using ELISA revealed that Nb44 specifically recognizes ICAM-1 and not the control protein BSA (**Figure 2C**). No signal was observed when the proteins were incubated with detection antibodies alone (**Figure 2C**: buffer).

### Nb44 displayed a slow dissociation rate and a high affinity to ICAM-1

**Figure 3:**
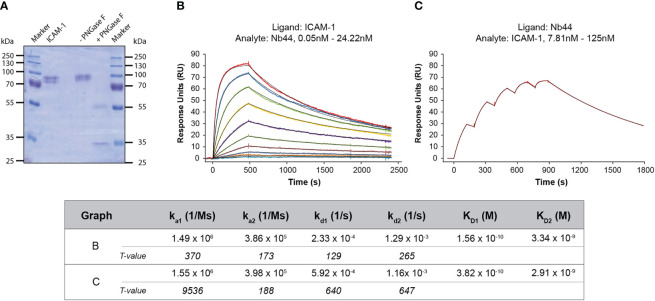
SPR-based study of the interaction between Nb44 and ICAM-1. **(A)** Separated and Coomassie Brilliant Blue-stained reaction mixtures of ICAM-1 deglycosylated by PNGase F. Lane 1: protein molecular mass marker (M), lane 2: ICAM-1, 1 µg, lane 3: ICAM-1 in reaction mixture without (-) PNGase F, lane 4: with (**+**) PNGase F, lane 5: protein molecular mass marker. Molecular masses are displayed on both margins in kDa. **(B)** Multi-cycle kinetics using ICAM-1 as ligand and Nb44 as analyte. Different colored lines correspond to different concentrations of Nb44. **(C)** Single-cycle kinetics with Nb44 as ligand (captured by anti-His-tag antibodies) and ICAM-1 as analyte. **(B, C)** Recorded curves (colored) were superimposed with calculated curves (black) according to a heterogeneous ligand model. Signal intensities (RU, y-axes) are shown versus time (in seconds, x-axes). Association and dissociation rate constants (k_a_, k_d_), dissociation constants (K_D_) and T values for each parameter are indicated. Displayed graphs are representatives of 4 **(B)** or 3 **(C)** independent experiments.

In order to investigate the kinetics between Nb44 and ICAM-1 we performed SPR-based assays. Employing the 1:1 binding model, we could not generate a satisfying fit to our recorded data. One plausible explanation is that the used ICAM-1 possessed distinct patterns of glycosylation, since it migrated as two separate bands in SDS-PAGE (**Figure 3A**). By digestion with the deglycosylase PNGase F, ICAM-1 was reduced to its predicted molecular mass of around 50 kDa (**Figure 3A**, + PNGase F). The band seen at 35 kDa corresponded to PNGase F (**Figure 3A**, + PNGase F). These experiments support the fact that the actually used ICAM-1 preparation is heterogeneous and consist of a protein with at least two different glycosylation patterns which show slightly different binding characteristics. Therefore, we used the heterogeneous ligand model for fitting the acquired data. We found that Nb44 quickly bound to immobilized ICAM-1 and formed stable complexes with dissociation rate constants (k_d_s) of 2.3*10^-4^/s and 1.3*10^-3^/s, which translated to an estimated half life time of around 50 and 10 minutes, respectively (**Figure 3B**). The resulting dissociation constants (K_D_s) were 1.6*10^-10^ M and 3.3*10^-9^ M, representing high affinity binding (**Figure 3B**). To strengthen our finding, we also performed an inverted assay using Nb44 as (immobilized) ligand and ICAM-1 as analyte. Employing again the heterogeneous ligand model, we obtained similar k_d_ values (5.9*10^-4^/s and 1.2*10^-3^/s) and K_D_ values (3.8*10^-10^ M and 2.9*10^-9^ M) (**Figure 3C**), confirming the high affinity and complex stability between Nb44 and ICAM-1.

### Nb44 recognized ICAM-1 on the cell surface of the human bronchial epithelial cell line 16HBE14o-

**Figure 4:**
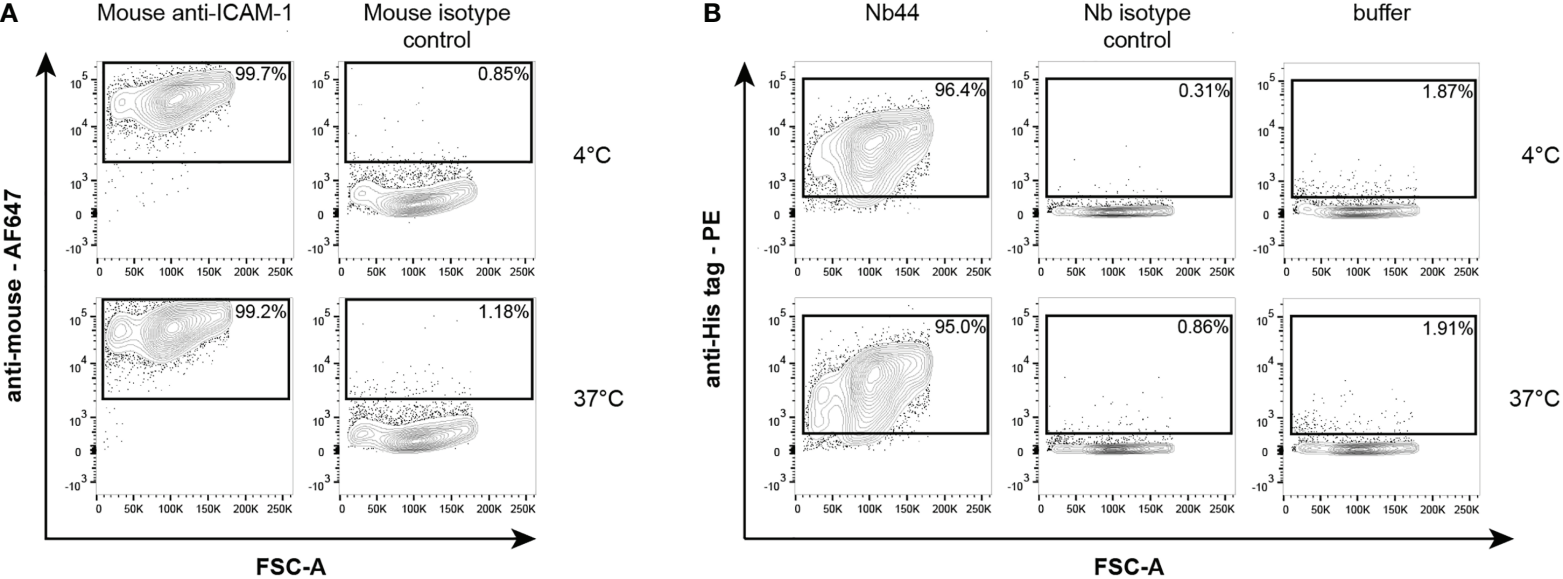
Flow cytometric analysis of mouse anti-ICAM-1 antibody **(A)** and Nb44 **(B)** binding to ICAM-1 expressed on 16HBE14o- cells. **(A)** Cells were incubated with a purchased mouse anti-ICAM-1 antibody or a mouse isotype control at 4°C (upper panels) or 37°C (lower panels), and subsequently stained with Alexa Fluor 467-conjugated anti-mouse antibody. Plots show forward scatter (FSC-A) (x-axes) versus anti-mouse Alexa Fluor 467 (y-axes). **(B)** Cells were incubated with Nb44 (left column), an isotype control (Bet v 1-specific Nb, middle column) or buffer alone (right column) at 4°C (upper panels) or 37°C (lower panels). Plots show forward scatter (FSC-A) (x-axes) versus PE-labeled anti-His tag antibody (y-axes). 16HBE14o- cells **(A, B)** were previously selected for alive cells by negative staining with eFluor^®^ 780 viability dye. Experiments shown in **(A, B)** were performed in triplicates and representative plots of three independent experiments are shown.

After showing that Nb44 bound to recombinant ICAM-1 with high affinity, we further wanted to test whether Nb44 recognized ICAM-1 expressed on the cell surface of human airway epithelial cells by flow cytometry. We first verified that the human bronchial epithelial cell line 16HBE14o- expressed ICAM-1 by staining with a commercially available mouse anti-ICAM-1 antibody, followed by an Alexa Fluor 647-conjugated anti-mouse antibody. We detected the anti-ICAM-1 antibody on 98.1 ± 2.2% and 97.2 ± 2.3% of live 16HBE14o- cells at 4°C and 37°C, respectively (**Figure 4A**). After confirming ICAM-1 expression, we incubated the cells with Nb44 or an isotype control (nanobody specific for Bet v 1) and stained with PE-labeled anti-His-tag antibody to detect bound Nb44. Nb44 recognized ICAM-1 on 97.2 ± 1.2% (4°C) and 96.7 ± 1.5% (37°C) of the live cell population (**Figure 4B**). Positive staining was neither observed for the isotype control (4°C: 0.3 ± 0.1%, 37°C: 0.8 ± 0.2%, **Figure 4B**), nor for the detection antibody alone (4°C: 1.0 ± 0.8%, 37°C: 1.0 ± 0.8%, **Figure 4B**: buffer). Percentages are expressed as mean values ± SD.

**Figure 5:**
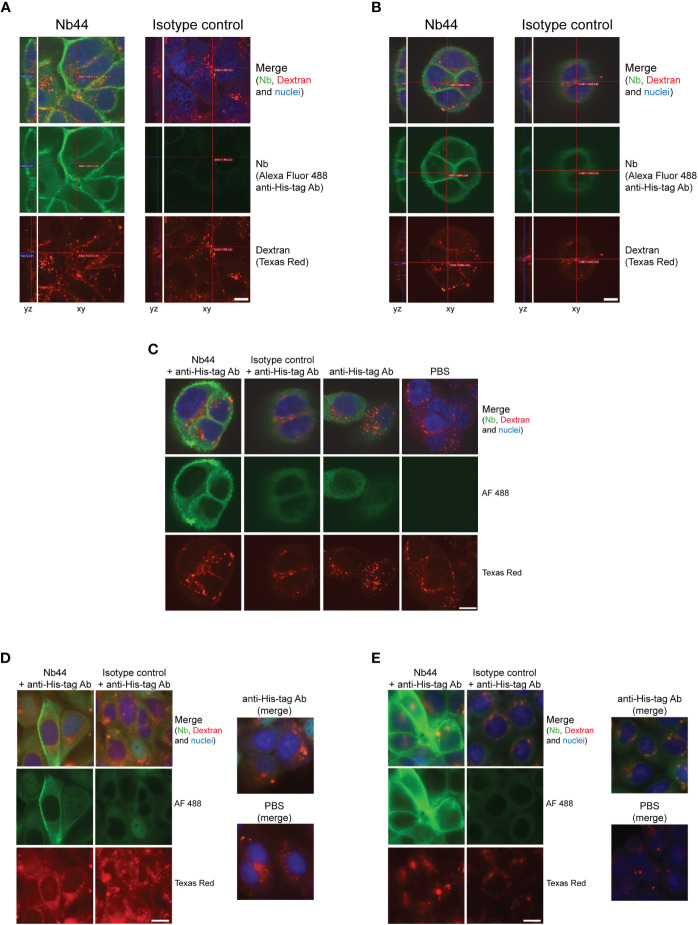
Nb44 binds to ICAM-1 on cell surfaces and is not internalized up to 24 hours. 16HBE14o- cells were simultaneously incubated with Texas Red-labeled Dextran (lysine-fixable) to label the endolysosomal system and Nb44 (left columns) or an isotype control (Bet v 1-specific Nb, right columns) for 30 minutes **(A–C)** 4 hours **(D)** or 24 hours **(E)**. After fixation with formaldehyde, cells were not permeabilized **(A)** or permeabilized with saponin **(B-E)**. Nanobodies bound to the plasma membrane were detected with Alexa Fluor 488-labeled anti-His-tag antibody (green). **(C)** For control purposes, cells were not only incubated with Nb44 and detection antibody (column 1), isotype control and detection antibody (column 2), but also with detection antibody alone (column 3) and PBS (column 4). **(A-C)** Confocal microscopic images showing yz-axes and xy-axes are representative of 3 independent experiments. **(D, E)** Representative wide field fluorescence microscopy images indicating Nb44 immobilization to the cell surface for 4 hours and 24 hours without internalization. Experiments were performed in duplicates. **(A-E)** Texas Red Dextran was present in all settings and cell nuclei were visualized with DRAQ5 (blue); scale bar is 10 µm.

To visualize the binding of Nb44 to and its immobilization at the cell surface of 16HBE14o- cells, we used immunofluorescence staining and fluorescence microscopy. First, cells were incubated simultaneously with Nb44 or an isotype control (Bet v 1-specific nanobody) and Texas Red-labeled Dextran for 30 minutes at 37°C. Thereafter, cells were fixed, but not permeabilized and nanobodies bound to the plasma membrane were detected with an Alexa Fluor 488-conjugated anti-His-tag antibody. While Nb44 was clearly localized on the cell surface, no binding was seen for the nanobody isotype control (**Figure 5A**). In contrast, Texas Red-labeled Dextran was internalized and marked the endolysosomal pathway (**Figure 5A**). In a second set of experiments, cells were incubated with Nb44 or the isotype control and Texas Red-labeled Dextran for 30 minutes, 4 hours and 24 hours at 37°C. After fixation, the cells were permeabilized to allow the detection antibody to access any internalized nanobody. We found that after 30 minutes as well as 4 hours and 24 hours, Nb44 was still immobilized at the plasma membrane exhibiting no colocalization with the labeled dextran that had entered the endolysosomal system (**Figures 5B–E**). Upon permeabilization, we observed some blurry fluorescence at excitation 488 nm in the cytoplasm for both Nb44 and the isotype control. Since binding of anti-His-tag antibodies to polyhistidine sequences in endogenous proteins in the cytoplasm has been described before (42, 43), we performed the following control experiment. Nanobodies were either present or omitted during uptake of the labeled dextran followed by incubation with the detection antibody. As additional control, the incubation steps with nanobodies and/or detection antibody were replaced with PBS. While incubation with PBS alone gave no background signal at excitation 488 nm, the Alexa Fluor 488-conjugated anti-His-tag antibody in the absence of nanobodies caused a similar cytoplasmic background fluorescence as observed in the presence of Nb44 or the isotype control, thus confirming that the cytoplasmic fluorescence signal was not derived from internalized nanobodies, but from endogenous proteins bound by the Fluor 488-conjugated anti-His-tag antibody (**Figure 5C**).

## Discussion

The transmembrane protein ICAM-1 plays a central role for many essential cellular functions both at the onset and resolution of pathologic conditions (44). Since ICAM-1 is highly upregulated in epithelial cells under inflammatory conditions where it primarily localizes to their apical surface (45), it has also been in the focus of treatment for allergic rhinoconjunctivitis (46, 47). Topically applied antihistamines induce downregulation of ICAM -1 expression on epithelial cells and thereby reduce eosinophil activation and allergen-induced inflammation (46, 47). However, using ICAM-1 as an anchor for bispecific antibodies for allergy treatment is a rather new concept (48). In a proof of principle study, antibody conjugates bispecific for ICAM-1 and pollen allergens were applied to capture and immobilize allergens *via* ICAM-1 to the apical surface of epithelial cells and thereby prevent allergen penetration through the epithelial barrier (21). Based on these observations we aimed to generate ICAM-1-specific nanobodies that combined characteristics needed for efficient binding to ICAM-1 and sustained attachment at the cell surface. The here described nanobody (Nb44) was obtained by immunizing a camel with recombinant ICAM-1 and subsequent screening of the resulting immune library. SPR-based affinity measurements showed high affinity binding of Nb44 to ICAM-1. The dissociation constants, calculated as the ratios of off/on-rate, were in the (sub)nanomolar range (10^-9^ to 10^-10^ M), which was in or above the range determined for other highly affine antibodies directed at ICAM-1 (49–51). Importantly, we measured slow off-rates of 10^-4^/s. Since our ultimate goal is to develop protective bispecific nanobody constructs to prevent allergen entry through the epithelium, the selected ICAM-1-specific nanobody should not only recognize ICAM-1 but also remain bound to maintain protection. Thus, a slow off-rate was a critical requirement for selecting the best nanobody candidate. Both SPR-based settings, with ICAM-1 either as ligand or analyte, yielded similar kinetic parameters. Considering all quality controls (standard error, T-value, multiple independent experiments) we report reliable data pointing to a highly affine nanobody with stable binding to ICAM-1.

It is well known that cell surface receptors, including ICAM-1, may be internalized after ligand binding. While this mechanism is used to shuttle drugs into or through endothelial cells to reach certain organs (33–35), it is not desired for our approach because we aim to immobilize the potentially attached allergens at the surface of epithelial cells. As demonstrated by flow cytometry Nb44 bound in a specific manner to ICAM-1 expressed on the human respiratory epithelial cell line 16HBE14o-. To explore potential internalization of Nb44, we performed immunofluorescence microscopy. In comparison to Texas Red-labelled Dextran that rapidly entered the endolysosomal system, Nb44 remained bound to ICAM-1 at the plasma membrane and did not localize in the endolysosomal pathway. For our initial experimental settings we chose 37°C temperature to imitate physiological conditions and a time period of 30 minutes. According to literature, endocytic uptake of multivalent anti-ICAM-1 ligands and antibodies occurs within 15 to 30 minutes, whereas monovalent ligands are hardly internalized or similar to the uptake/recycling pathway of ICAM-1 in the absence of ligands (36, 37, 52–54). To detect potential uptake over an extended period of time, nanobodies were incubated with 16HBE14o- cells for 4 h and 24 h. However, even after 24 hours topically applied Nb44 remained localized on the cell surface, demonstrating that it was not internalized to the cytoplasm. Since our selected nanobody is monovalent, these results confirmed data reported in earlier studies postulating that multimeric ligands are necessary for clustering ICAM-1 to be internalized (35, 54). However, it remains to be investigated if prospective bispecific formats, *i.e.*, ICAM-1/allergen-specific nanobodies will be taken up by epithelial cells while bound to ICAM-1. The fact that nanobodies do not *per se* cross-link two or more antigens, *e.g*., ICAM-1 expressed on the cell surface encourages efforts to engineer heterodimers with allergen-specific nanobodies.

Since the presence of seasonal allergens is limited to several weeks every year, it is tempting to develop nanobody-based approaches for topical allergy treatment. Nasal and ocular epithelial cell surfaces are exposed to allergen quantities in the range of ng/day (55), thus stopping allergen penetration with rather low amounts of specific nanobodies topically applied to target organs of allergic rhinoconjunctivitis seems feasible. Increasing numbers of nanobodies specific for a variety of clinically relevant allergens are becoming available (27–30, 56, 57). It should therefore be possible to develop allergen/ICAM-1 nanobody constructs for diverse respiratory allergens that can be flexibly combined to protect patients against several different allergen sources. Due to the fact that allergic individuals are first exposed to airborne allergens *via* inhalation or by contact with the conjunctiva, it is envisaged to administer bispecific nanobodies to the nose and eyes and eventually to the lung in order to treat respiratory and conjunctival allergic symptoms. However, further *in vivo* investigations are required to evaluate if topically administered nanobodies prevent allergen-induced respiratory inflammation in a clinically relevant manner.

Allergy still represents a new application field for nanobodies, yet in recent years a number of allergen-specific nanobodies have been developed to detect the respective allergens in food sources (27–29). Furthermore, a nanobody capable of abrogating allergen-mediated basophil activation by mimicking CD23, the low affinity receptor for IgE, thus disrupting the bond between IgE and FcϵRI, was generated and suggested to act more efficiently than Omalizumab (58). Very recently, nanobody-IgE formats were described to act as surrogates in hymenoptera venom allergy and the authors speculated that formats consisting of blocking nanobodies and IgG antibodies could be used in interventional studies (56). These data are in line with our findings that allergen-specific or anti-IgE nanobodies could be effective for allergy treatment (38, 57, 58).

Their unique characteristics such as chemical stability, single domain organization and the simplicity of reformatting render nanobodies as versatile tools for the development of various nanobody-based drugs. We here report the generation and evaluation of an ICAM-1 specific nanobody. Due to its stable binding behavior we identified Nb44 as an appropriate candidate for anchoring nanobody constructs to ICAM-1. We envision that Nb44 could be used to engineer allergen/ICAM-1-specific heterodimers to develop bispecific nanobodies for topical treatments of pollen allergy.

## Data availability statement

The original contributions presented in the study are publicly available. The nucleotide sequence data can be found in the GenBank repository under accession ON950079.

## Ethics statement

The animal study was undertaken in accordance to the National Standard of the Russian Federation GOST R 53434-2009 and with approval from the Commission on Bioethics (formed on May 3, 2017) on February 2, 2018 (registration number 17) in the Severtsov Institute of Problems of Ecology and Evolution.

## Author contributions

SF and ST designed the experiments, IZ, TI, MZ, MR, IE, OG, JK, SV-M, CL, CW and ST performed experiments, IZ, MZ, IE, AD, JE-D, ST and SF analyzed data. IZ and SF wrote the manuscript. All authors contributed to the article and approved the submitted version.

## Funding

This study was supported by Austria Science Fund (FWF) grants I3946-B33 and F4607, by the Russian Foundation for Basic Research (RFBR) grant 18-515-14003 and by the Country of Lower Austria’s funded Danube Allergy research cluster.

## Acknowledgments

We thank Professor Dieter Gruenert from the University of California, San Francisco, Mt Zion Cancer Center, for kindly providing the cell line 16HBE14o-, Professor Serge Muyldermans from Vrije Universiteit Brussel, Belgium, for kindly providing the plasmid pHEN6, and Dr. Pia Gattinger from Medical University of Vienna, Institute of Pathophysiology and Allergy Research for providing the enzyme Peptide-N-Glycosidase F and her theoretical assistance for the deglycosylation experiment. Additionally we thank Dr. Erika Garner-Spitzer for manuscript proof-reading and editing.

## Conflict of interest

AD is employed by Cytiva GmbH. CL reports personal fees from Thermofisher, outside the submitted work. JE-D reports grants and personal fees from Astrazeneca, personal fees from Sanofi and GSK, outside the submitted work.

The remaining authors declare that the research was conducted in the absence of any commercial or financial relationships that could be construed as potential conflict of interest.

## Publisher’s note

All claims expressed in this article are solely those of the authors and do not necessarily represent those of their affiliated organizations, or those of the publisher, the editors and the reviewers. Any product that may be evaluated in this article, or claim that may be made by its manufacturer, is not guaranteed or endorsed by the publisher.

## Abbreviations

BSA, Bovine Serum Albumin; FcϵRI, high affinity receptor for IgE; HBS, Hepes-buffered Saline; HRP, Horse-Radish Peroxidase; ICAM-1, Intercellular Adhesion Molecule 1; Nb, Nanobody; PBMC, Peripheral Blood Mononuclear Cell; PBS, Phosphate-buffered Saline; PBST, Phosphate-buffered Saline + 0.05% Tween 20; SPR, Surface Plasmon Resonance.
