# Supplementary material for: Generation of high affinity ICAM-1-specific nanobodies and evaluation of their suitability for allergy treatment
Source: Front Immunol. 2022 Nov 9;13:1022418. doi: 10.3389/fimmu.2022.1022418 (PMC9682242; doi:10.3389/fimmu.2022.1022418)
Supplement: Supplementary file 1 [file Table_1.docx]

**Supplemental Data**

**Generation of high affinity ICAM-1-specific nanobodies and evaluation of their suitability for allergy treatment**

Ines Zettl^1^, Tatiana Ivanova^2^, Mohammed Zghaebi^3^, Marina V. Rutovskaya^2,4^, Isabella Ellinger^5^, Oksana Goryainova^2^, Jessica Kollárová^1^, Sergio Villazala-Merino^3^, Christian Lupinek^1^, Christina Weichwald^1^, Anja Drescher^6^, Julia Eckl-Dorna^3^, Sergei V. Tillib^2*^, Sabine Flicker^1^*

^1^Division of Immunopathology, Institute for Pathophysiology and Allergy Research, Center for Pathophysiology, Infectiology and Immunology, Medical University of Vienna, Vienna, Austria

^2^Institute of Gene Biology, Russian Academy of Sciences, Moscow, Russia

^3^Department of Otorhinolaryngology, Medical University of Vienna, Vienna, Austria

^4^A.N.Severtsov Institute of Ecology and Evolution, Russian Academy of Sciences, Moscow, Russia

^5^Division of Cellular and Molecular Pathophysiology, Institute for Pathophysiology and Allergy Research, Center for Pathophysiology, Infectiology and Immunology, Medical University of Vienna, Vienna, Austria

^6^Cytiva Europe GmbH, Freiburg, Germany

*Co-corresponding and co-last authors: Sabine Flicker, Division of Immunopathology, Institute for Pathophysiology and Allergy Research, Center for Pathophysiology, Infectiology and Immunology, Medical University of Vienna, Vienna, Austria; Phone: +43-1-40400-51150; Fax: +43-1-40400-51300; Email: sabine.flicker@meduniwien.ac.at and Sergei V. Tillib, Institute of Gene Biology, Russian Academy of Sciences, Moscow, Russia; Phone: +7 499-135-2201; Fax: +7 (499) 135-41-05; Email: tillib@genebiology.ru

**Table S1:** Antibodies, endocytic marker and fluorescent dyes used in Western blot (WB), ELISA, flow cytometry (FC) and immunofluorescence microscopy (IFM).

| **Antibodies, endocytic marker or fluorescent dyes** | **Conjugate** | **Company** | **Clone** | **Cat. no** | **used in** |
| --- | --- | --- | --- | --- | --- |
| Anti-HA-tag | HRP | Sigma-Aldrich | HA-7 | H6533-1VL | WB, ELISA |
| Mouse anti-ICAM-1 | unconjugated | LSBio | 15.2 | LS-C134488 | FC |
| Mouse IgG1,  κ isotype control | unconjugated | BD Biosciences | MOPC-21 | 555746 | FC |
| Anti-His-tag | PE | Biolegend | J095G46 | 362603 | FC |
| Anti-mouse IgG | Alexa Fluor 647 | Invitrogen | polyclonal | A21235 | FC |
| Fixable viability dye eFluor 780 | unconjugated | ThermoFisher Scientific |  | 65-0865-14 | FC |
| Anti-His-tag | Alexa Fluor 488 | Invitrogen | 4E3D10H2/E3 | MA1-135-A488 | IFM |
| Dextran,70 kDa | Texas Red | ThermoFisher Scientific |  | D1864 | IFM |
| DRAQ5 | unconjugated | ThermoFisher Scientific |  | 62254 | IFM |
